# Long-term high-fat diet increases glymphatic activity in the hypothalamus in mice

**DOI:** 10.1038/s41598-023-30630-y

**Published:** 2023-03-13

**Authors:** Christine Delle, Neža Cankar, Christian Digebjerg Holgersson, Helle Hvorup Knudsen, Elise Schiøler Nielsen, Celia Kjaerby, Yuki Mori, Maiken Nedergaard, Pia Weikop

**Affiliations:** 1 Center for Translational Neuromedicine, Faculty of Medical and Health Sciences, University of Copenhagen, Blegdamsvej 3B, 2200 Copenhagen N, Denmark; 2 Center for Translational Neuromedicine, University of Rochester Medical School, Elmwood Avenue 601, Rochester, NY 14642 USA

**Keywords:** Neuroscience, Diseases

## Abstract

Obesity affects millions of people worldwide and is associated with an increased risk of cognitive decline. The glymphatic system is a brain-wide metabolic waste clearance system, dysfunction of which is linked to dementia. We herein examined glymphatic transport in mice with long-term obesity induced by a high-fat diet for 10 months. The obese mice developed hypertension and elevated heart rate, neuroinflammation and gliosis, but not apparent systemic inflammation. Surprisingly, glymphatic inflow was globally unaffected by the high-fat diet except for the hypothalamus, which displayed increased influx and elevated AQP4 vascular polarization compared to the normal weight control group. We propose that a long-term high-fat diet induced metabolic alteration of hypothalamic neurons and neuroinflammation, which in turn enhanced glymphatic clearance in the effected brain region.

## Introduction

The prevalence of obesity is rising yearly in children and adolescents around the world^1^. Obesity is the leading risk factor for developing hypertension^2^, type II diabetes^1^ and is further linked to increased risk for developing certain neurodegenerative diseases^3–5^. It is well-established that the brain glymphatic system is suppressed in a broad range of neurodegenerative diseases, including Alzheimer’s disease^6^, which may suggest a causal pathway proceeding from obesity.

Being devoid of lymphatic vessels, the brain utilizes a unique brain-wide fluid system for export of metabolic waste, called the glymphatic system^7^. In this fluid transport pathway, periarterial cerebrospinal fluid (CSF) influx into the parenchyma is facilitated by the water channel aquaporin-4 (AQP4), which is highly expressed in astrocytic endfeet plastered around blood vessels. Extracellular fluid carrying metabolic waste products is exported along the perivenous space and cranial and spinal nerves before being collected by meningeal and cervical lymph vessels and returned to the general circulation^8^.

Glymphatic fluid flow is driven in part by arterial pulsation^9–11^ such that its transport correlates inversely with heart rate^11,12^. The flow rate and arterial pulsation are both compromised by hypertension, which is a common complication of obesity^2^. The development of obesity-related hypertension is linked to increased lipolysis and elevated secretion of adipokines such as angiotensin II and renin that adversely impact vascular tone^13^. Furthermore, the adipokine leptin, which normally functions to suppress appetite, is chronically elevated in obesity, and overstimulates the sympathetic nervous system, thereby increasing the risk for hypertension^14^. Hypertension is further promoted by impaired endothelial cell function arising due to the chronic metabolic, oxidative, and inflammatory stresses evoked by obesity^15–17^. In addition, chronic fluid accumulation due to sodium retention within tissue^18,19^ may create a systemic fluid disbalance^20^ that could possibly affect glymphatic function. Indeed, impaired glymphatic clearance was previously described in a spontaneously hypertensive rat model^21^. However, no studies have investigated the effect of obesity on glymphatic flow. We hypothesized that obesity-associated hypertension would impair glymphatic activity in mice. To test this hypothesis, we measured markers of glymphatic function in mice with obesity and hypertension evoked by long-term high-fat diet (HFD)^22,23^ as compared to a lean control group.

## Results

### High-fat diet induces obesity, elevated blood pressure, increased heart rate, and hyperglycemia

**Figure 1:**
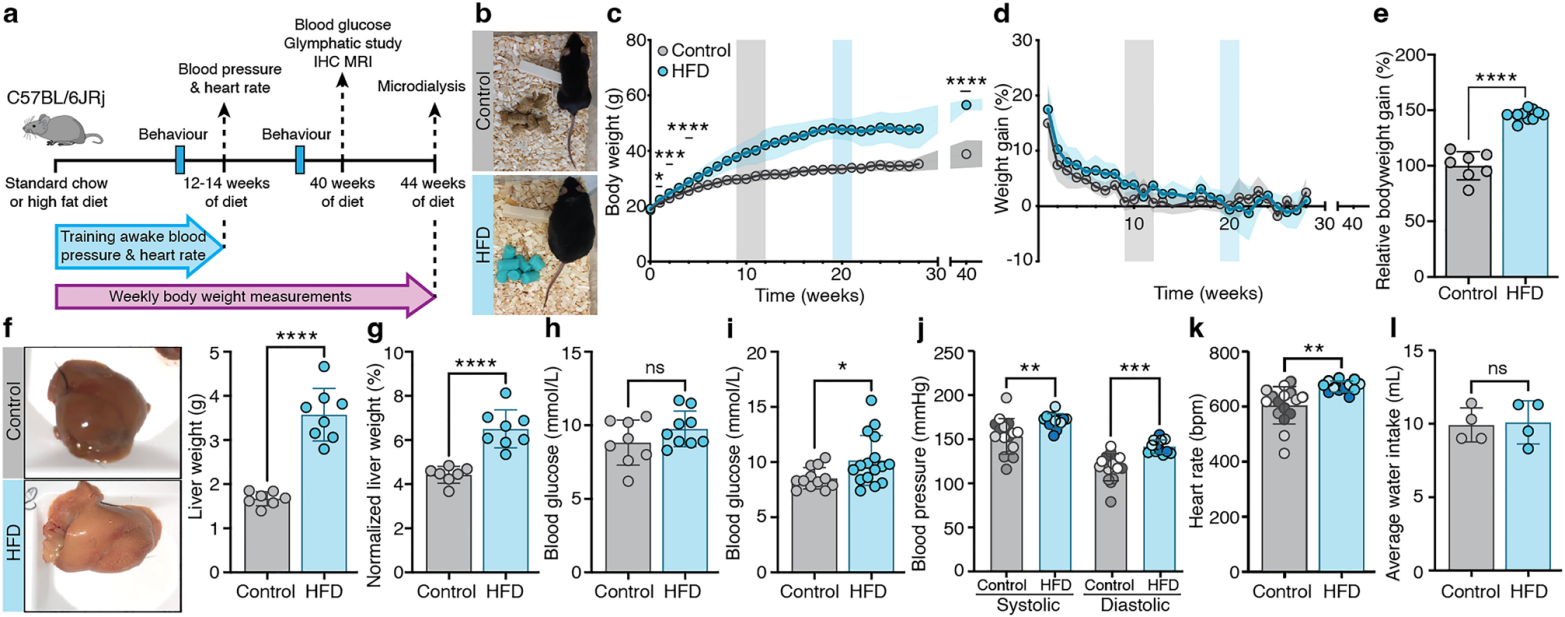
Long-term high-fat diet (HFD) causes severe obesity in C57BL/6JRj mice. (**a**) Schematic timeline of experimental setup. (**b**) Representative control (standard chow fed) and high-fat diet (HFD) mouse freely moving in the home cage (40 weeks of diet). (**c**) Body weight development (n = 9–18 per time point). Grey and blue shaded areas display the time of body weight adaptation. (**d**) Weight gain per timepoint compared to previous weekly body weight (n = 9–18 mice per time point). (**e**) Weight gain compared after 40 weeks of diet, normalized to average control weight. (**f**) Representative liver images and total liver weight in grams (g) (n = 6–8). (**g**) Liver weights normalized to the animal's body weight (n = 7–8). (**h**) Blood glucose measurements in awake non-fasted (n = 8–9) and (**i**) fasted mice (n = 12–16). (**j**) Systolic and diastolic blood pressure measured in awake mice (2-way ANOVA) and (**k**) heart rate in beats per minute (bpm) measured in awake mice (n (control) = 4 mice, 4 measurements per mouse; n (obese) = 3 mice, 4 measurements per mouse, color coded by mouse). (**l**) Averaged water intake in grams (g) per mouse per day (n = 4 cages, water consumption averaged per mouse). Unless otherwise indicated all graphs are analyzed via unpaired t-test. Graphs created with GraphPad software (version 9.0, https://www.graphpad.com/scientific-software/prism/). Schematic created in Adobe Illustrator 2022 (version 26.3.1, https://www.adobe.com/products/illustrator/free-trial-download.html) by Dan Xue.

We first studied relevant phenotypic changes to confirm the robustness of our obesity model. Starting at 6 weeks of age, groups of C57BL/6JRj mice received either normal chow or a HFD for the next 44 weeks to induce long-term obesity (Fig. 1a, b). HFD mice showed relatively elevated body weight within two weeks compared to controls (HFD: 22.36 g ± 1.37 g, control: 21.27 g ± 1.36 g; *p* = 0.0228, Fig. 1c). The mean body weight of the HFD group continued to rise over the course of the study (Fig. 1c), exceeding by 46 ± 4.9% the weight of chow-fed controls at 40 weeks after the start of HFD (Fig. 1e).

Notably, the control mice reached a plateau of maximal body weight after 9–12 weeks of standard chow diet (15–18 weeks of age) (Fig. 1c,d; gray shaded area), in contrast to the continuous weight gain until 20 weeks of HFD (26 weeks of age) (Fig. 1c,d; blue shaded area). Drastic morphological changes in HFD mouse livers manifested after 40 weeks, when the liver tissue appeared pale and the organ weight had nearly doubled (HFD: 3.57 g ± 0.59 g; control: 1.67 g ± 0.16 g; *p* < 0.0001, Fig. 1f). Thus, ratio of liver to body weight was significantly higher in HFD mice as compared with controls (HFD: 6.50 ± 0.86%; control: 4.42 ± 0.38%; *p* < 0.0001, Fig. 1g). At 40 weeks into the diet, there was no significant increase in blood glucose baseline levels (HFD: 9.76 ± 1.21 Mmol/L; control: 8.83 ± 1.53 Mmol/L; *p* = 0.18, Fig. 1h), but upon 7 h of fasting the HFD mice showed mild hyperglycemia (HFD: 10.15 ± 2.26 Mmol/L; control: 8.53 ± 0.96 Mmol/L; *p* = 0.0289, Fig. 1i). HFD also increased systolic and diastolic blood pressure (systolic: HFD: 171 ± 8 mmHg; control: 153 ± 20 mmHg; *p* = 0.0047; diastolic: HFD: 142 ± 8 mmHg; control: 118 ± 15 mmHg; *p* = 0.0002, Fig. 1j) after 12–14 weeks, with increased heart rate in trained mice (HFD: 673 ± 20 bpm; control: 605 ± 68 bpm; *p* = 0.0023, Fig. 1k, see Suppl. Fig. S1 for blood pressure training). The water intake of HFD mice, which could be a sign of pre-diabetes, remained unchanged (Fig. 1l).

### High-fat diet impairs behavior but not overall mobility

**Figure 2:**
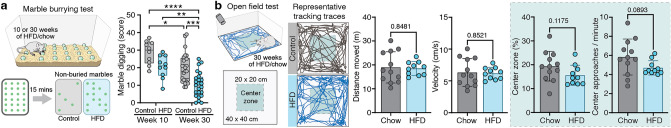
Long-term high-fat diet (HFD) impairs behavior but not mobility of mice. (**a**) Schematic of marble burying test and quantification of marble burying score (2-way ANOVA, n = 10 (10 weeks) and 24–25 (30 weeks)). (**b**) Schematic of open field test followed by quantification of moved distance (meter) (left panel), velocity (cm/s) (second panel), time spent in the center zone (percent) (green shaded left panel) and center approaches per minute (green shaded right panel). n = 10 – 12. Unless otherwise indicated all graphs are analyzed via unpaired t-test. Graphs created with GraphPad software (version 9.0, https://www.graphpad.com/scientific-software/prism/). Schematics created in Adobe Illustrator 2022, https://www.adobe.com/products/illustrator/free-trial-download.html) by Dan Xue. Representative tracking traces created in MATLAB R2022a and Adobe Illustrator (version 26.3.1, https://www.adobe.com/products/illustrator/free-trial-download.html).

Long-term HFD has been reported to alter behavior^24,25^. We first assessed the natural digging behavior of mice using the marble burying test^26^ (Fig. 2a). At 10 weeks HFD mice already showed a trend towards reduced burying activity (21 ± 7; control: 29 ± 7, *p* = 0.1151), and after 30 weeks of diet, the HFD mice demonstrated significantly reduced activity compared to age-matched controls (10 ± 7; control: 20 ± 9; *p* = 0.0004). Interestingly, control mice also revealed reduced digging activity after 30 weeks of standard chow compared to their 10-week time point (10-week: 29 ± 7; 30-week: 20 ± 9; *p* = 0.0131). Yet, the decline in this activity due to diet was greater for HFD mice compared to the standard chow group (10-week: 21 ± 7; 30-week: 10 ± 7; *p* = 0.0038). An open field test at 30 weeks showed no group differences in the distance traveled or mean velocity (Fig. 2b). Interestingly, there was a trend for HFD mice approaching less often the center area of the open field arena.

### Long-term high-fat diet accelerates hypothalamic glymphatic activity

**Figure 3:**
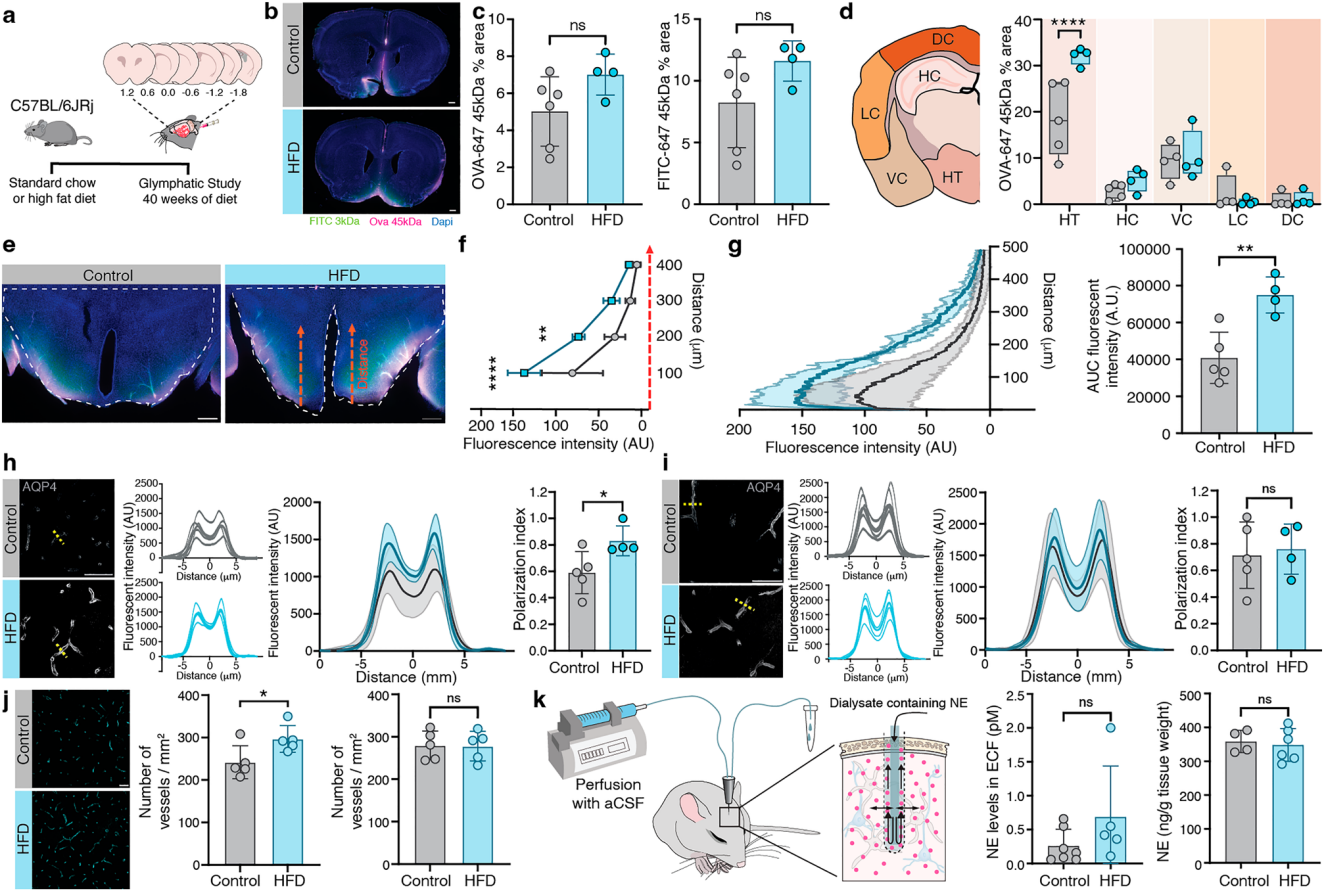
Increased glymphatic activity and AQP4 polarization in hypothalamus after 40 weeks of high-fat diet. (**a**) Intracisternal CSF tracer injection and brain sectioning. (**b**) Representative coronal brain sections (bregma 1.2 mm) with tracer signal. Scale bars: 200 µm. (**c**) Quantification of 45 kDa Ovalbumin-647 tracer (n = 4–5, ns = 0.0947) and 3 kDa FITC-tracer (n = 4–5, ns = 0.127) for 6 brain slices per animal shown as % total area. (**d**) Schematic of regional analysis for tracer fluorescent signal in hypothalamus region (HT), hippocampus (HC), ventral, lateral and dorsal cortex (VC, LC, DC respectively) at bregma -1.80 and corresponding regional quantification of the 45 kDa Ovalbumin-647 tracer (n = 4–5, 2-way ANOVA, **** < 0.0001). (**e**) Representative hypothalamus slice showing region of interest (bregma -1.80), red arrows = line ROIs used for tracer penetration analysis, assigned as a distance. Line ROIs positioned perpendicular to the brain surface. (**f**) Quantification of tracer intensity in the hypothalamus (AU = arbitary units). Increased tracer signal in HFD mice at 100 and 200 µm (2-way ANOVA, **** < 0.0001, ** = 0.0024). (n = 4–5 mice). (**g**) Left; Representative hypothalamic fluorescent signal of ovalbumin (45 kDa). Right; total fluorescent tracer signal shown as area under the curve (n = 4–5, ** = 0.0044), AU = arbitary units. (**h**, **i**) Representative AQP4 staining with line ROIs drawn across blood vessels (yellow dotted lines) in (**h**) hypothalamus and (**i**) hippocampus. Scale bars: 50 µm. Line ROI traces were plotted for each animal. Lines represent average fluorescent trace of 12–36 blood vessels per mouse (n = 4–5). Summarized traces are shown as group averages ± SD. Right: Polarization index of AQP4 in the (**h**) hypothalamus (* = 0.0388) and (**i**) hippocampus (ns = 0.7677) (n = 4–5). (**j**) Representative AQP4 staining in hypothalamus for quantification of vessel density in (left) hypothalamus (* = 0.0399) and (right) hippocampus (ns = 0.9457) (n = 5). (**k**) Illustration of microdialysis sampling of extracellular fluid (ECF) in hippocampus and quantification of norepinephrine (NE) levels in extracellular fluid (left) and hippocampus homogenates (right) (n = 5–7; ECF: ns = 0.1810, homogenates: ns = 0.7183). Graphs show mean ± SD. Unpaired t-tests if not otherwise indicated. Graphs created with GraphPad software (version 9.0, https://www.graphpad.com/scientific-software/prism/). Analysis of line ROI traces analyzed with ImageJ software (version 2.1.0/1.53c, https://imagej.nih.gov/ij/download.html). Schematics created in Adobe Illustrator 2022 (version 26.3.1, https://www.adobe.com/products/illustrator/free-trial-download.html) by Dan Xue.

To examine CSF transport, we injected a mixture of two tracers with different molecular sizes (3 and 45 kDa) into the cisterna magna and allowed circulation for 30 min much as previously described^6,7,10,12,27,28^, comparing mice after 40 weeks HFD diet with controls (Fig. 3a). The brains were harvested and six selected coronal vibratome sections of 100 µm thickness (1.2 to −1.8 from bregma) where microscopically examined for tracer infiltration (Fig. 3b). Interestingly, we found no brain-wide changes in tracer signal for either the small or large CSF tracers (OVA: HFD: 7.0 ± 1.1 a.u.; control: 5.0 ± 1.8 a.u., FITC: HFD: 11.6 ± 1.6 a.u., control: 8.2 ± 3.7 a.u., Fig. 3c). Additionally, there were no changes of total brain volume between groups according to magnet resonance imaging (MRI) in vivo (Suppl. Table S1).

Regional analysis of tracer distribution in dorsal, lateral, and ventral cortex, hippocampus and hypothalamus depicted no difference in cortex or hippocampus between the groups (Fig. 3d). Strikingly however, we noted increased tracer distribution area (%) in the hypothalamus of HFD mice (2-way ANOVA, main effect of region, F(4, 32) = 82.1, *p* < 0.0001, Fig. 3d) and increased tracer penetration into the hypothalamic parenchyma at distances 100 and 200 µm from the brain surface (2-way ANOVA, main effect of distance, F(3, 28) = 63.0, *p* < 0.0001, Fig. 3f).

### Long-term high-fat diet increases AQP4 vascular polarization and density in the hypothalamus

AQP4 is expressed on astrocytic endfeet surrounding blood vessels, where it facilitates glymphatic fluid flow^28^. Coronal brain sections were next immunolabeled for AQP4 and its vascular polarization was evaluated by placing line ROIs through the vessel structures to map the profile of AQP4 signal^28^ in hypothalamus and hippocampus (Fig. 3h,i). The AQP4 polarization index was significantly increased around blood vessels in the hypothalamus of HFD group compared to aged-matched controls (HFD: 0.83 ± 0.11, control: 0.59 ± 0.16, Fig. 3h) but not in hippocampus (HFD: 0.76 ± 0.19, controls: 0.71 ± 0.25, Fig. 3i). We next assessed the vascular density in hypothalamus and hippocampus based on the AQP4 immune marker. There was a significant increase with HFD in the density of blood vessels per square mm in hypothalamus (HFD: 296.8 ± 31.46 control: 242.1 ± 38.77, Fig. 3j). In contrast, we found no difference between groups in vessel density in the hippocampus (HFD: 278.0 ± 34.85 control: 279.5 ± 33.92, Fig. 3j).

### Consistent norepinephrine levels support unaltered brain wide glymphatic activity

Earlier studies have shown that norepinephrine (NE) is an inhibitor of glymphatic flow^29^. Lower levels of NE generally promote glymphatic flow while higher levels decrease it^29^. Quantification of NE in hippocampus of HFD and standard chow mice showed no group difference in extracellular fluid collected via microdialysis (HFD: 0.67 ± 0.75, control: 0.26 ±0.24) or in hippocampus homogenates (HFD: 348.0 ± 48.47, control: 358.5 ± 32.97, Fig. 3k).

### Long-term high-fat diet is linked to neuroinflammation, but not systemic inflammation

**Figure 4:**
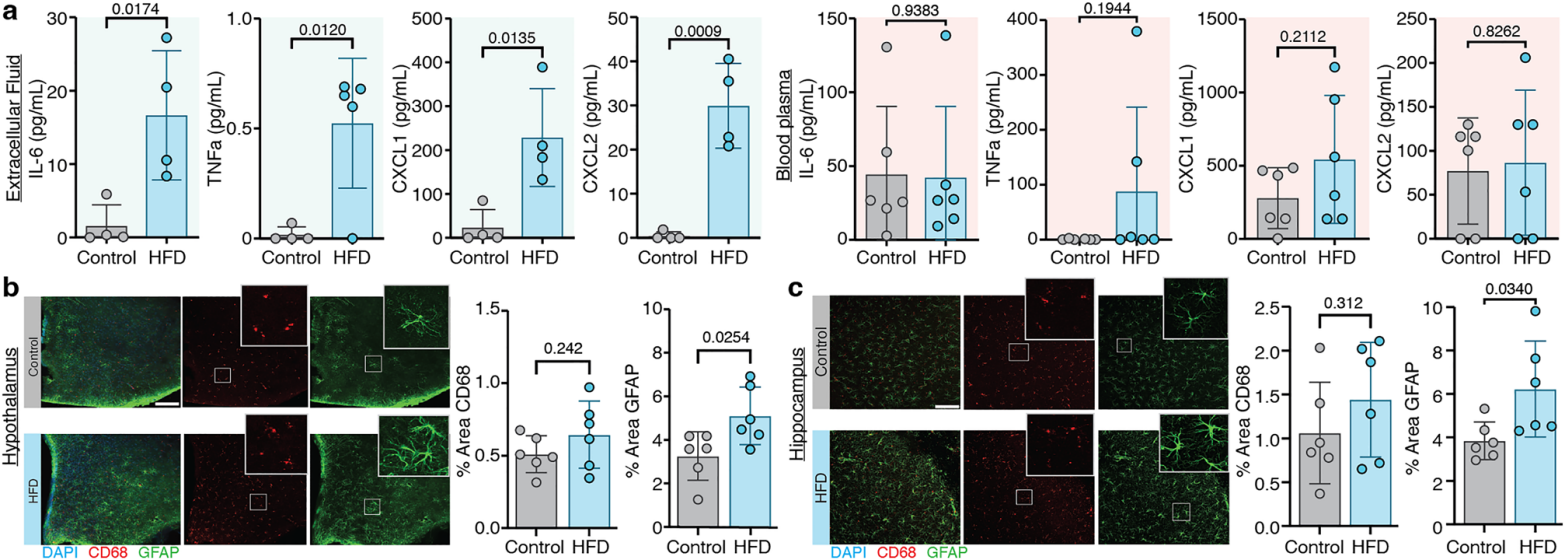
Chronic obesity induces brain inflammation but not peripheral inflammation. (**a**) Cytokine and chemokine profiles of extracellular fluid (green shaded panels) and blood plasma (red shaded panels) for IL-6, TNFα, CXCL1 and CXCL2 of HFD or control mice (n = 4–6; unpaired t-test, IL-6 * = 0.0174; TNFα * = 0.0120; CXCL1 * = 0.0135; CXCL2 *** = 0.009). (**b**, **c**) Immunohistochemistry of coronal brain slices of HFD and control mice after 44 weeks of diet. Representative images of (**b**) hypothalamus and (**c**) hippocampus regions for the microglial marker CD68 and astrocytic marker GFAP. Quantification of % total area stained for microglial CD68 and astrocytic GFAP marker in (**b**) hippocampus and (**c**) hypothalamus. Scale bars: 50 µm. Graphs shows mean ± SD (n = 6, unpaired t-test, hypothalamus % area GFAP * = 0.0254, hippocampus % area GFAP * = 0.0340). Graphs created with GraphPad software (version 9.0, https://www.graphpad.com/scientific-software/prism/). Figure design in Adobe Illustrator 2022 (version 26.3.1, https://www.adobe.com/products/illustrator/free-trial-download.html) by Dan Xue.

Previous studies reported low-grade neuroinflammation and systemic inflammation induced by obesity^30–32^. Since the hippocampus and hypothalamus are affected by obesity-related neuroinflammation in humans and rodents, we focused on these two regions^31–33^ evated in the median eminence (ME), supraoptic nucleus (SON) and periventricular nucleus (PEVN) in both sexes of diabetic rats. We measured the cytokine profile in blood plasma and in samples of extracellular fluid collected via microdialysis in the hippocampus in mice receiving HFD or standard chow diet for 44 weeks. Elevated extracellular fluid concentrations of the cytokines IL-6 (HFD: 16.65 ± 8.81 pg/mL, control: 1.58 ± 2.88 pg/mL) and TNFα (HFD: 0.52 ± 0.29 pg/mL, control: 0.018 ± 0.035 pg/mL) and the chemokines CXCL1 (HFD: 228.8 ± 111.6 pg/mL, control: 23.16 ± 41.29 pg/mL) and CXCL2 (HFD: 29.91 ± 9.60 pg/mL, control: 0.46 ± 0.93 pg/mL) were detected in HFD mice (Fig. 4a; left). In contrast, blood plasma of HFD mice showed no elevation of any cytokines/chemokines compared to the control group (Fig. 4a; right).

Of note, MRI relaxometry revealed no differences between HFD and control mice for T1 and T2 relaxation times (Suppl. Table S1), which indicated no apparent global neuroinflammation. On the other hand, T1 and T2 relaxation times were both reduced in the ventricular system (T1: HFD: 2277 ± 56.60 ms, control: 2577 ± 90.45 ms, *p* = 0.0182; T2: HFD: 59.57 ± 2.956 ms, control: 70.82 ± 3.816 ms, *p* = 0.0420, Suppl. Table S1).

To evaluate cellular markers of glial reactivity, we analyzed the CD68-positive (microglial cells) and GFAP-positive (astrocytic cells) in vibratome sections. Interestingly, there were no detectable differences in total fluorescence coverage of the microglial marker (Fig. 4b,c) either in hypothalamus (HFD: 0.48 ± 0.15%area, control: 0.44 ± 0.13%area) or hippocampus (HFD: 1.44 ± 0.65%area, control: 1.06 ± 0.58%area). However, we found an increased GFAP fluorescence coverage in hypothalamus (HFD: 4.99 ± 1.62% area, control: 2.97 ± 1.43%area) and hippocampus (HFD: 6.22 ± 2.20% area, control: 3.85 ± 0.87%area), indicating that astrogliosis was induced by chronic HFD in both regions (Fig. 4b,c). The observed differences in astrocytic but not microglial reactivity could indicate that astrocytes are the key drivers of diet-induced neuroinflammation. Overall, neuroinflammation, but not systemic inflammation, was evident in this mouse model of chronic obesity.

## Discussion

We here utilized a HFD mouse model to study the impact of obesity on the glymphatic system. Mice receiving the HFD developed severe obesity as well as hypertension, increased heart rate, and signs of neuroinflammation but no systemic inflammation within the blood compartment (Figs. 1, 4). Elevated fasting blood glucose levels in HFD mice was also noted (Fig. 1i), which indicates the development of insulin resistance, a hallmark of type II diabetes^34^. The marble burying test revealed progressively impaired behavior of HFD mice (Fig. 2a), while open field assessment showed no difference in total walked distance, but a trend for obese mice to approach the center area less frequently (Fig. 2b). Previous studies have shown cognitive decline in both human and rodents with obesity^24,25^, and obese humans show an increased risk of developing Alzheimer’s disease^35^. Although control mice showed declining performance with age in the marble burying test, the HFD group exhibited accelerated age-related behavioral impairment (Fig. 2a).

Surprisingly, we detected no changes in global glymphatic flow, even though the HFD mice developed a severe obesity phenotype (Fig. 1a–c). The structural MRI examination displayed no changes in brain volume (Suppl. Table S1), but T1 and T2 relaxation times in the ventricles were reduced in HFD. An earlier study has shown declining ventricular T1 and T2 relaxation times in association with increased CSF protein levels in patients with type II diabetes^36^.

The rate of glymphatic fluid transport is inversely proportional to heart rate and vascular pulsation^9–12^. A previous study in spontaneously hypertensive rats (SHR) revealed significant glymphatic impairments to dynamic contrast-enhanced MRI imaging^21^. However, the SHR model was obtained by selective inbreeding, resulting in considerably greater increases in systolic blood pressure (relative increase of 60–70 mmHg) as compared to our HFD-induced obesity mice. In agreement with the current study, Weisbrod et al. reported comparable relative changes in systolic blood pressure (an increase of 10–20 mmHg) in mice after 7–12 months of high-fat-high-sugar (HFHS) diet. Furthermore, the same study showed that arterial stiffness and hypertension were reversable within 2 weeks after changing HFHS diet back to standard chow^37^.

Our study is the first to provide insight into glymphatic flow after HFD lasting 10 months, a duration far exceeding that in most previous experimental models^38–40^. Hence, the lacking effect of HFD on glymphatic flow (Fig. 3a–c) may reflect adaptations to maintain brain homeostasis. Adaption to diet-induced obesity was previously reported to occur for the microvasculature of the blood brain barrier via suppression of metabolic alterations^41^. Although a high-fat diet induces pro-inflammatory cytokine release and astrogliosis, such changes appear to be time-dependent and can subside over time^42^. Neuroprotective adaptations in glymphatic flow may also develop if mice are chronically exposed to a HFD. The present finding of unaltered glymphatic activity in HFD mice is further supported by the absence of changes in the NE level in the extracellular fluid and hippocampal homogenates (Fig. 3k). As a key modulator of the glymphatic system, pharmacological manipulation of NE impacts glymphatic activity^29^. Reduction of NE levels resulted in increased glymphatic flow, mirroring the onset of glymphatic activity observed during sleep or with certain anesthetics^29^. Thus, the lack of significant changes in brain volume and NE level changes supports the absence of brain-wide glymphatic alterations in response to HFD.

Nonetheless, we uncovered focally increased glymphatic activity, revealed by elevated total tracer uptake and increased tracer penetration at the hypothalamic surface of HFD mice (Fig. 3d–g). Among many functions, the hypothalamus controls energy homeostasis and regulates appetite^43^. Chronic HFD increased excitatory synaptic transmission of hypothalamic neurons^44^. Whereas cytoskeletal proteomic changes and increased synaptic plasticity were reported in HFD mice, hypothalamic energy metabolism may be elevated, giving rise to increased demand for extracellular metabolite removal^45^. In keeping with this, we observed increased vascular density in the hypothalamic region of HFD mice (Fig. 3j). Angiogenesis was previously observed in hypothalamus of type 2 diabetic patients and after exposure to high-caloric diet in mice^46^. Particularly striking is the present observation of increased AQP4 polarization in hypothalamic cerebral vasculature of the HFD group (Fig. 3h,i). In accordance with regional glymphatic tracer influx analysis, the AQP4 polarization was identified only in hypothalamus and not in hippocampus (Fig. 3h,i). Glymphatic transport is driven by AQP4 vascular polarization, and AQP4 knockout (KO) mice exhibit lower CSF tracer influx^27^. We speculate that long-term HFD increased the need for fluid trafficking in the hypothalamus, which was underpinned by locally enhanced AQP4 polarization and increased vessel density, and a resultant increase in glymphatic tracer influx^24,44^.

Obesity impairs lymphatic function and shrinks lymph nodes^47^. An earlier study in HFD mice indicated reduced fluid uptake and transport via peripheral lymphatic vessels ^47^. Lymphatic alterations are driven by chronic inflammation, immune cell activation, and decreased vascular endothelial growth factor (VEGF) expression^24,48^. Cytokines IL-6, TNFα, IL-10 and chemokine CXCL1 are the key plasma inflammatory markers in obesity^49–51^. We analyzed several pro-inflammatory cytokines and chemokines, including mediators that can signal a Th1 and Th2 response, via a multiplex panel. Most parameters were undetectable despite a very low detection limit and measurable markers showed no differences between groups which indicated no apparent systemic inflammation within the blood compartment of the HFD group (Fig. 4a). There, however, was a trend towards increased TNFα, IL-10, IL-5, IL-17α, CXCL1 and CXCL2 levels, which was driven by a subpopulation of HFD mice. Yet, that observation may be in accordance with previous reports of low-grade peripheral inflammation^52,53^, which was localized within the adipose tissue, rather than in the blood circulation^54^. It is possible that peripheral proinflammatory mediators could directly affect brain perivascular fluid transport by signaling across the BBB^55^.

We saw increased levels of pro-inflammatory markers in extracellular fluid of hippocampus, which was confirmed by cellular markers of glia reactivity in our HFD model. In particular, there were elevated brain levels of the cytokines IL-6 and TNFα and the chemokines CXCL1 and CXCL2 (Fig. 3a). Obesity-related insulin and leptin resistance is reported in hypothalamus^30,56^, together with neuroinflammation^31,32^ and gliosis^48^. Mutual interactions of metabolic hypothalamic adaptation and neuroinflammatory responses are presently under investigation^56–58^, with the implication that chronic HFD gliosis paired with TNFα secretion may arise after disruption of neuronal metabolic homeostasis. As such, an obesity-related shift in brain energy and metabolic expenditure may promote glymphatic flow changes in the hypothalamus.

Since microdialysis sampling in the hypothalamus region is technically challenging, we undertook additional immuno-staining in hippocampus and hypothalamus. In both regions, there was a significant increase in the astrocytic marker GFAP (Fig. 4b,c). Reactive astrocytes that produce pro-inflammatory mediators have been reported to arise with long term HFD in humans and rodent models^59–61^. On the other hand, the microglial marker CD68 did not exhibit any changes in our model (Fig. 4b,c). While astrocyte reactivity develops gradually, the microglial cells contribute to pathometabolic changes principally by acute secretion of pro-inflammatory signaling molecules^62^. Indeed, microglial activation in hypothalamus was previously shown to be evident as early as 6 h after HFD consumption^58^. While our study is limited to the use of CD68 as single microglia marker, it still indicates the absence of a reactive phenotype. CD68 particularly shows microglia activation since it labels lysosomes^63^ and was in a recent study clearly shown to be upregulated in mice after a long-term 9-months HFD^64^.Therefore, we suppose that astrocytes may be the dominant factor in the hypothalamic alterations caused in our chronic model of diet-induced obesity.

In summary, we provide the first evidence for regionally accelerated glymphatic activity in the hypothalamus of mice after long-term HFD, in the absence of major changes in glymphatic flow in other brain regions. This could reflect an increased demand for hypothalamic fluid exchange and metabolic waste removal in diet-induced obesity in mice.

## Methods

### Animals and diets

6-week-old male C57BL/6JRj mice (Javier, France) were housed in groups of five and received either a standard chow diet (SAFE, Germany, D30) or a high-fat diet (Research Diets, Inc., USA, D12492) for up to 44 weeks to induce obesity (Suppl. Table S2). Food and water were provided ad libitum*.* Mice were housed in a 12/12 light/dark cycle (lights on 7am) at room temperature (22 ± 2 °C) and a relative humidity of 55 ± 10%. Experimental data was collected at 10, 30, 40 and 44 weeks after chow/HFD.

All experiments were performed at the Center for Translational Neuromedicine at the University of Copenhagen with national approval from the Danish Animal Inspectorate and local approval by the Department of Animal Experimental Medicine of the University of Copenhagen. The study was carried out in compliance with the ARRIVE guidelines.

### Monitoring of body weight, water intake and blood glucose

Mice were weighed weekly to monitor weight gain over the entire experimental period of 40 weeks. To estimate potential changes in water consumption, we weighed the water bottles before and after refill of control and obese mice home cages in week 40. Individual water intake was calculated as the average consumption per day divided by the number of mice per cage and plotted by day. To examine whether the high-fat diet increased blood glucose baseline levels in obese mice, we measured the blood glucose in week 40 in awake non-fasting mice. Here, blood was obtained from the tail tip after needle puncture and measured with a BAYER Countour apparatus and test stripes (Ascensia). Blood glucose measurements were also performed at 7 h of food deprivation (mice received fresh cages), to compare the response of blood glucose levels to fasting in control and obese mice.

### Marble burying and open field test

A marble burying test was performed as previously described^26^ to compare general activity and natural burying behavior of obese and control mice. A thick layer of fresh bedding was provided in an open standard housing cage (Tecniplast 1284, Blue line type 2) and 28 marbles were spread evenly across the surface. Each mouse was allowed 15 min to explore the cage and was then returned to its home cage. A blinded researcher afterwards entered the room and counted the number of marbles fully buried (score = 2 points), half buried (score = 1 point), and not buried/unmoved (score = 0 points). This simple scoring system to quantify the natural activity behavior of control and obese mice was plotted to compare activity between both groups. To test whether the results of the marble burying test were influenced by body weight and general mobility, we performed an open-field test, where the mice were allowed to explore an empty arena (40 × 40 cm) with a defined center area (20 × 20 cm). Video recording was performed using Synapse (Tucker Davis Technologies) and Basler cameras. Afterwards, the total distance, velocity, and time spent and number of entries to the center area were analyzed using EthoVision XT.

### Blood pressure and heart rate monitoring

To examine systolic and diastolic blood pressure and heart rate in awake control and obese mice, we used a non-invasive tail-cuff method (CODA® Monitor, Kent Scientific). Mice were trained weekly for up to 12 weeks for the procedure prior to conducting the experimental measurements (Suppl. Fig. S1). Mice that did not habituate well and showed visual signs of distress were excluded from this analysis of recordings. For monitoring blood pressure and heart rate, mice were placed into a CODA® animal holder of appropriate size (small: 0–25 g mice; medium: 25–50 g mice; large: 50–75 g mice) and habituated 15–20 min prior to recordings. A CODA X-Small Occlusion Cuff and VPR Cuff were placed on the base of the tail and 25 measurements taken per mouse (Mode: Mouse; cycle interval 10 s, deflation time 15 s). The collected measurements were checked for stable readouts throughout the measurement. Mice that showed fluctuations indicating stress or cases of failure due to movements of the animal were excluded. The final four measurements were used for further analysis if stable readouts were obtained throughout the recording. A minimum of three mice per group mice were used for this analysis.

### Intracisternal CSF tracer injection

To investigate glymphatic dynamics, tracers were injected intracisternal as previously described^11,27^. In brief, anesthetized mice (ketamine (100 mg/kg) and xylazine (20 mg/kg)) were head-fixed in a stereotactic frame and a dental needle (SOPIRA^®^Carpule^®^30G-0.30 × 12 metric, Kulzer GmbH, Germany) attached to a P10 tubing (Scandidact ApS, Denmark) was inserted into the cisterna magna. Over a 10-min period 10 µl of mixture of 45 kDa Alexa Fluor 647-conjugated ovalbumin (O34784, ThermoFisher Scientific) and 3 kDa fluorescein-conjugated dextran (FITC, ThermoFisher, D3305) (both to equal parts, Suppl. Table S3) at a final 1% concentration (w/v, in artificial cerebral spinal fluid (155 mM NaCl, 3.5 mM KCl, 1 mM CaCl_2_, 1 mM MgCl_2_, and 2 mM NaH_2_PO_4_, pH 7.4, 300 mOsm)) was infused at a rate of 1 µl/min utilizing a 100 µL syringe (81,020, Hamilton Bonaduz AG, Bonaduz, Switzerland) mounted on a motorized pump (Havard Apparatus Pump 11 Elite (USA). Physiological body temperature was maintained with a heating pad (Rodent Warmer X2, Stoelting Europe, Ireland) placed below the body. All glymphatic-related experiments were conducted in the sleeping period of the animals between 9 am and 5 pm. Tracer was allowed to circulate 30 min before the animals were sacrificed.

### Analysis of tracer distribution in the brain

After tracer circulation, brains were harvested and fixed by immersion for 24 h in 4% paraformaldehyde in phosphate-buffered saline. Coronal vibratome (Leica VT1200 S) slices (100 μm) were cut and mounted (Prolong® Gold Antifade Mountant, P36934, Thermo Fisher Scientific). Tracer distribution in the brain was acquired on whole-slice brain sections using a standard fluorescence microscope (Nikon ECLIPSE Ni-E) and a digital camera (Mono-Camera Nikon DS-Fi3) controlled by an imaging software (NIS-Elements Imaging software AR 4.60.00) at constant exposure times throughout the study. Images were analyzed in ImageJ software (version 2.1.0/1.53c), keeping a uniform threshold (pixel intensity 50 out of 255) and subtracting background fluorescence. The thresholded area was expressed as percentage of the entire brain slice area. To obtain a single biological measure per animal, the mean tracer area coverage for seven brain slices was averaged. For regional analysis of hypothalamus, ventral cortex, lateral cortex and dorsal cortex, the section –1.8 mm from bregma was divided into corresponding regions of interests. Regional tracer analysis of hippocampus was performed on the section –2.4 mm from bregma, due to larger hippocampal area.

### In vivo magnet resonance imaging

After 40 weeks of either chow or HFD, mice underwent MRI under isoflurane anesthesia (3% induction, and 1–1.5% maintenance) in a 1/1 mixture of air/oxygen. The body temperature was maintained at 37 ± 0.5 °C with a thermostatically controlled waterbed and monitored, along with the respiratory monitoring by an MR-compatible remote monitoring system (SA Instruments, NY, USA). MRI was performed in a 9.4 T animal scanner (BioSpec 94/30 USR, Paravision 6.0.1 software, Bruker BioSpin, Ettlingen, Germany). Imaging was acquired using a 1H cryogenically-cooled quadrature-resonator Tx/Rx coil (CryoProbe) and 240 mT/m gradient coil (BGA-12S, Bruker). The MRI protocol comprised a T2-weighted rapid acquisition with relaxation enhancement (RARE) sequence with the following settings: repetition time (TR) = 7000 ms, effective echo time (TE) = 35 ms, RAREfactor = 16, number of average (NA) = 6, field of view (FOV) = 14.4 mm × 14.4 mm, matrix = 192 × 192, slice thickness = 0.3 mm, bandwidth (BW) = 75 kHz. Fifty coronal slices were acquired to cover the entire brain. Total experiment time = 8 min for T2-weighted imaging. For T1 relaxometry, rapid acquisition with relaxation enhancement at variable TR (RARE-VTR) was used for saturation recovery sequence. The imaging parameters were TE = 10 ms, TR array = 27, 30, 40, 60, 80,100, 120, 200, 300, 400, 600, 800, 1000, 2000, 4000, and 8000 ms, RARE factor = 4, NA = 2, slice thickness = 1 mm, FOV = 14.4 mm × 14.4 mm, matrix = 128 × 128, BW = 100 kHz, and total experiment time = 19 min for each T1-map. In addition, T2 relaxation time was measured with multi-slice multiecho (MSME) sequence. The imaging parameters were: TR = 3000 ms, number of echoes = 40 with echo spacing = 7 ms, NA = 2, slice thickness = 1 mm, FOV = 14.4 mm × 14.4 mm, matrix = 128 × 128, BW = 100 kHz, and total experiment time = 13 min for each T2-map. T1- and T2-maps were generated using an image sequence analysis tool package (Paravision 6.0.1, Bruker), which uses a fit function:

$${\text{For the T1}} - {\text{map}}:{\text{ M}}\left( {\text{t}} \right) \, = {\text{ A }} + {\text{ M}}0 \, * \, \left( {{1 } - {\text{ exp}}\left( {{\text{t}}/{\text{T1}}} \right)} \right),$$

where A = absolute bias, M0 = the equilibrium magnetization, and T1 = longitudinal relaxation time.

$${\text{For the T2}} - {\text{map}}:{\text{ y }} = {\text{ A }} + {\text{ C }}*{\text{ exp}}\left( { - {\text{t}}/{\text{T2}}} \right),$$

where A = absolute bias, C = signal intensity, and T2 = transverse relaxation time.

MRI analysis was performed in ITK-SNAP (version 3.8.0)^65^. The image bias field was removed using Advanced Normalization Tools (ANTs N4 bias correction)^66,67^. The brain volume was automatically segmented by using the region-growing function with ITK-SNAP. In addition, pixel intensity-factorized semi-automatic thresholding was performed to segment hippocampus and the lateral ventricle in each hemisphere. The volume measurement of each compartment was performed in ITK-SNAP.

### Immunohistochemistry

Coronal brain slices (100 µm) were stained for the glial markers GFAP, the water channel AQP4 and the microglial marker CD68 (Suppl. Table S3). Tissue sections were blocked at room temperature for two hours in normal goat serum (5% in PBS, and 0.3% Triton-X 100 (Sigma Aldrich). The primary antibodies were diluted in the same blocking solution and the tissue incubated overnight at 4 °C on an orbital shaker. After a PBS wash, primary antibodies were conjugated with the appropriate secondary antibodies coupled to fluorophores (Alexa Fluor, 1:500; Invitrogen™ Molecular Probes™; Thermo Fisher Scientific), in PBS for 2 h at room temperature while protected from light. After a 5 min nuclear counterstaining with DAPI (4’,6-diamidino-2-phenylindole, Thermo Fisher Scientific, 62,248, 1:1000), tissue sections were mounted on glass slides with Prolong Gold Antifade Reagent (Invitrogen/Thermo Fisher Scientific, Carlsbad, CA, USA). Images were then taken with a confocal microscope (Nikon Eclipse Ti, Tokyo, Japan) using Plan Fluor 20X/0.75, 40X/1.30 and 60x/1.40 oil objectives. Obtained images were analyzed using FIJI/ImageJ for MAC (ver. 2.3.0/1.53q).

### Quantification of immuno-stained brain sections

We acquired 50 µm confocal z-stacks of CD68 and GFAP markers in the hypothalamus and hippocampus regions (at 40 × magnification). The maximum projection intensities were then assessed in FIJI/ImageJ software. The immunohistochemical signal was thresholded for each marker and the signal expressed as percent area coverage. For each animal we acquired 3 confocal images in each brain region and averaged the signal to obtain a single biological replicate.

For assessing AQP4 polarization towards blood vessels, we performed an analysis much as previously described^28^. Herein, multiple line ROIs were drawn across the blood vessel structures in hypothalamus. To get a representative trace for each mouse, 12–36 blood vessels per animal were analyzed. Obtained traces were aligned using cross-correlation, with the first trace serving as a reference. Polarization index was calculated by subtracting 5 µm of background signal to the vascular peak fluorescent value. All values were subsequently normalized to the highest fluorescent. Vessel density analysis was done by creating a mask on the thresholded image with the AQP4 signal using MaxEntropy. The Fiji/ImageJ tool Analyse Particles was used to count number of blood vessels per mm^2^. Only the particles bigger than 20 µm^2^ were included for quantification.

### Microdialysis

To investigate NE levels in diet-induced obesity mice underwent microdialysis surgery as previously described^68^. Microdialysis of NE was performed in ventral hippocampus (A/P: − 3.0 mm, M/L: −3.0 mm and D/V: −3.0 mm), whereas samples for K^+^ measurement were collected in striatum (AP: + 1.0 mm, M/L: + 1.5 mm, D/V: −2.0 mm) In brief, the microdialysis guide canula was implanted stereotaxically at 24 h prior to sampling. Under isoflurane anesthesia (induction 4%, maintenance 1.6–2%) mice were mounted to a stereotactic frame and received lidocaine (0.1 mL, 1 mg/kg; Accord) subcutaneously at the site of the scalp incision and trepanation, whereupon the microdialysis guide canula was implanted (CMA 8 Microdialysis Probe Guide, Harvard Apparatus). After wound closure, mice were housed in their home cage and received carprofen (5 mg/kg; ScanVet) for pain relief. Microdialysis sampling was performed using a syringe (CMA syringe type I, Harvard Apparatus) mounted on a pump (CMA 402 Syringe Pump, Harvard Apparatus). The syringe filled with aCSF (119 mM NaCl, 3.5 mM KCl, 1.0 mM CaCl_2_, 0.8 mM MgCl_2_, HEPES 10 mM, pH 7.2, dissolved in dH_2_O) was connected to a 6 kDa cut-off microdialysis probe (CMA 7 CMA Microdialysis AB, Sweden). Sampling was performed in the natural sleeping phase of the mice (approximately 10:00–14:30). Constant flow rate (0.1 µL/min) was applied throughout the experiment. To minimize variation of sampling results due to technical differences, an obese and a control mouse were always yoked to a shared injection pump. Collected ISF samples were immediately snap frozen and stored at −80 °C until analysis for NE content by high-performance liquid chromatography with electrochemical detection (HPLC-ECD). After termination of the experiments, the mice were decapitated, and the brains were quickly removed and stored at −80 °C. The correct placement of microdialysis probes was histologically verified. Only results from mice with correct probe placements are reported. The contralateral brain hemisphere was used for preparation of tissue homogenates. The striatum and hippocampus were dissected and homogenized in 150 µL 0.1 M perchloride acid and thereafter analyzed for NE as described above.

### Cytokine analysis of microdialysis and plasma samples

To assess hippocampal neuroinflammatory changes due to diet-induced obesity, we performed microdialysis sampling of cytokines after 44 weeks of diet. Here we perfused high cut-off microdialysis probes (CMA 8 High Cut-Off 100 kDa, Harvard Apparatus) with aCSF + 3% BSA mixture. Sampling was performed during animal resting phase (9 am to 3 pm) at a constant flow rate of 0.8 µL/min. Mice were killed by decapitation and trunk blood collected in sterile EDTA-coated 1.5 mL centrifuge tubes and centrifuged at 1000 g for 10 min at 4 °C. The blood plasma supernatant was separated and snap frozen on dry ice. Cytokine levels in microdialysis samples and plasma were then analyzed using the Multiplex Laser Bead Assay by Eve Technologies (MDHSTC18, Calgary, Canada) as previously described y^69^. The multiplexing technology was performed using the Bio-Plex TM 200 system (Bio-Rad Laboratories, Inc., Hercules, CA, USA) and Mouse-High-Sensitivity-18-Plex Discovery Assay panel (Millipore, St. Charles, MO, USA) to quantify different biomarkers in the same samples, which were measured in duplicate. Here, we present only data of cytokines that were detectable by the assay and displayed significant differences between the groups (IL-6, TNFα, CXCL1, CXCL2, Fig. 4). Detectible cytokines without significant differences are attached in Supplementary Fig. S2.

### Statistical analysis

Statistical analysis was performed using GraphPad Prism version 9.0 (GraphPad Software). For comparison of the mean values following a normal distribution, we used an unpaired t-test when analyzing two groups. For datasets with more than two groups, we applied a 2-wayANOVA to compare the means, followed by Šídák's multiple comparisons test. The significance level of all performed analysis in this study was a = 0.05. If not otherwise stated, all values are expressed as mean ± SD.

### Ethical approval

The animal experimental procedures were approved by the local research ethics committee (Department of Experimental Medicine, University of Copenhagen) and conducted in accordance with the Danish Animal Experiments Inspectorate.

## Supplementary Information

Supplementary Information.

## Data Availability

All data needed to evaluate the conclusions drawn in this paper are included in this paper. Additional data is available from the corresponding author upon reasonable request.
