# Supplementary material for: Long-term high-fat diet increases glymphatic activity in the hypothalamus in mice
Source: Sci Rep. 2023 Mar 13;13:4137. doi: 10.1038/s41598-023-30630-y (PMC10011420; doi:10.1038/s41598-023-30630-y)
Supplement: Supplementary file 1 — Supplementary Information. [file 41598_2023_30630_MOESM1_ESM.docx]

**Long-term high-fat diet increases glymphatic activity in the hypothalamus in mice**

Christine Delle^1+^, Neža Cankar^1+^, Christian Digebjerg Holgersson^1^, Helle Hvorup Knudsen^1^, Elise Schiøler Nielsen^1^, Celia Kjaerby^1^, Yuki Mori^1^, Maiken Nedergaard^1,2*^& Pia Weikop^1^

^1^Center for Translational Neuromedicine, Faculty of Medical and Health Sciences, University of Copenhagen, Blegdamsvej 3B, 2200 Copenhagen N, Denmark.

^2^Center for Translational Neuromedicine, University of Rochester Medical School, Elmwood Avenue 601, Rochester, NY 14642, USA.

**_
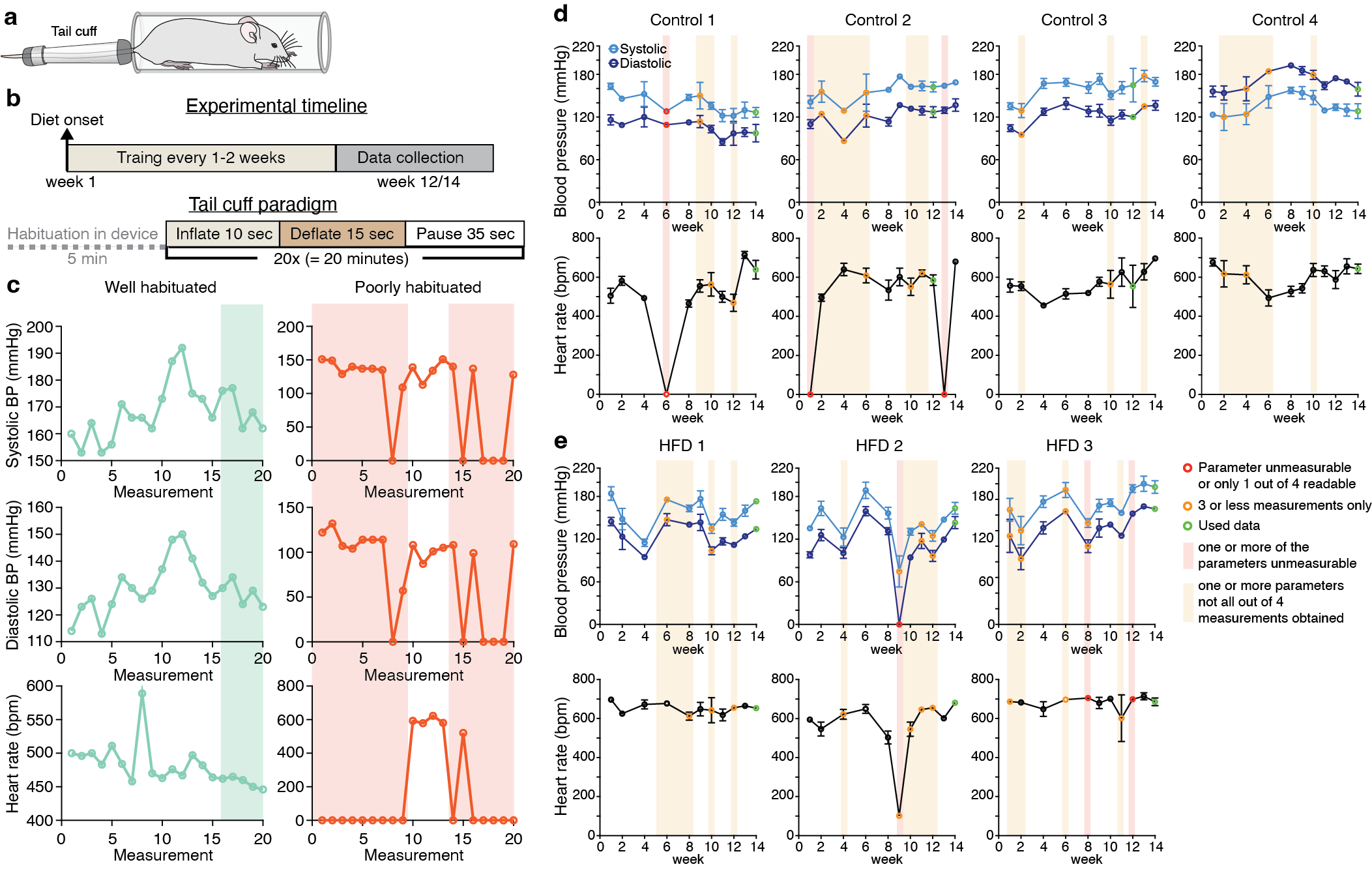
_**

**Supplementary Figure S1: Blood pressure and heart rate monitoring.** a) Schematic of an awake mouse positioned in a non-invasive tail-cuff device to measure blood pressure and heart rate. b) Experimental timeline (top) and paradigm for one round of measurements (bottom). c) Example of a single round of 20 minutes measurement in a well-habituated animal left) and yet unhabituated animal (right). Green shaded area shows the last 4 minutes of the 20-minute procedure with well acclimated heart rate, indicating that the end phase of the experimental design is most suitable to collect reliable blood pressure and heart rate data. One value per minute per parameter was collected. Red shaded area indicates time points of non-readable heart rate indicating animal movement and stress. d + e) Development of blood pressure and heart rate over the duration of 14 weeks shown for 4 control (d) and 3 HFD (e) animals. Mean ± SD (n = 4 measurements/animal/timepoint). Red dot: Parameter unmeasurable for all 4 measurements and defined as 0 or only 1 out of 4 measurements obtained. Orange dot: 3 or less out of 4 measurements readable. Green dot: Data of this time point used for analysis in figure 1. Red shaded area: one or more parameters unmeasurable or inaccurate measurements due to animal stress or movement. Yellow shaded area: one or more parameters not optimally obtained. Graphs created with GraphPad software (version 9.0, <https://www.graphpad.com/scientific-software/prism/>). Schematic created in Adobe Illustrator 2022 (version 26.3.1, [https://www.adobe.com/products/illustrator/free-trial-download.html](https://www.adobe.com/products/illustrator/free-trial-download.html)) by Dan Xue.

**
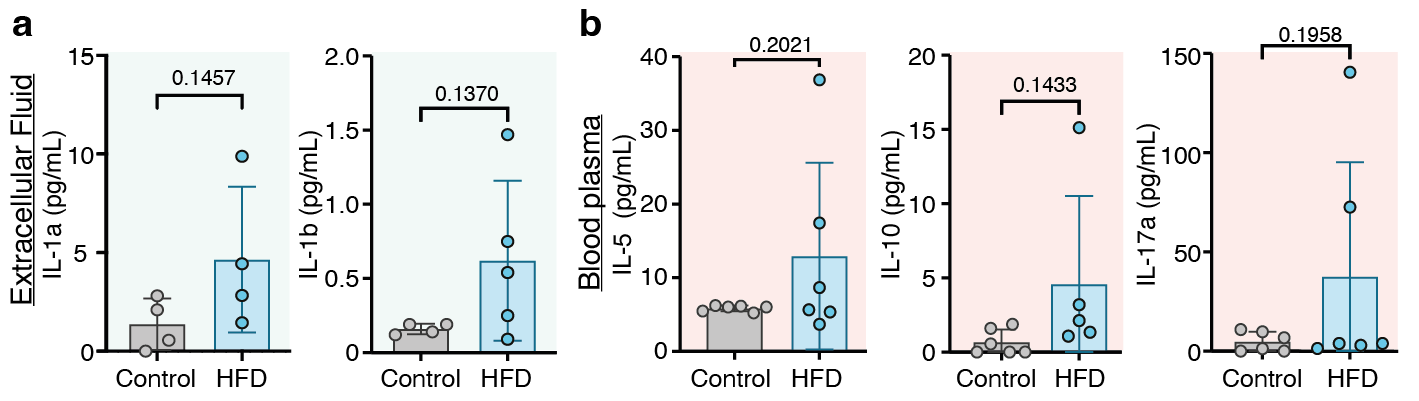
**

**Supplementary Figure S2: Non-significant extracellular fluid and blood plasma cytokine profiles.** a) Cytokines IL-1a and IL-1b, from extracellular fluid (n = 4-6; unpaired t-test, IL-1a: ns = 0.1457, IL-1b: ns = 0.1370). b) Plasma cytokines IL-5, IL-10 and IL-17a (n = 4-6; unpaired t-test, IL-5: ns = 0.2021, IL-10: ns = 0.1433, IL-17a: ns = 0.1958). All graphs show mean ± SD. Other cytokines were below detection levels in ISF or blood plasma and therefore not included. Graphs created with GraphPad software (version 9.0, <https://www.graphpad.com/scientific-software/prism/>).

**Supplementary Table S1: MRI of mice receiving 40-weeks of HFD or standard chow diet.** Table depicts calculated volumes and T1 and T2 relaxation times of several brain regions (red: hippocampus, purple: lateral ventricle, green: thalamus, yellow: hypothalamus). Blue shading in the table indicates parameters showing differences between control and HFD fed mice (p < 0.05). All values show mean ± SD (unpaired t-tests for normally distributed data and Mann-Whitney test in case of not normally distributed data, n = 6). MRI analysis, thresholding and volume measurement performed in ITK-SNAP (version 3.8.0, <http://www.itksnap.org/>). T1- and T2-maps were generated using an image sequence analysis tool package (Paravision 6.0.1, Bruker).

| 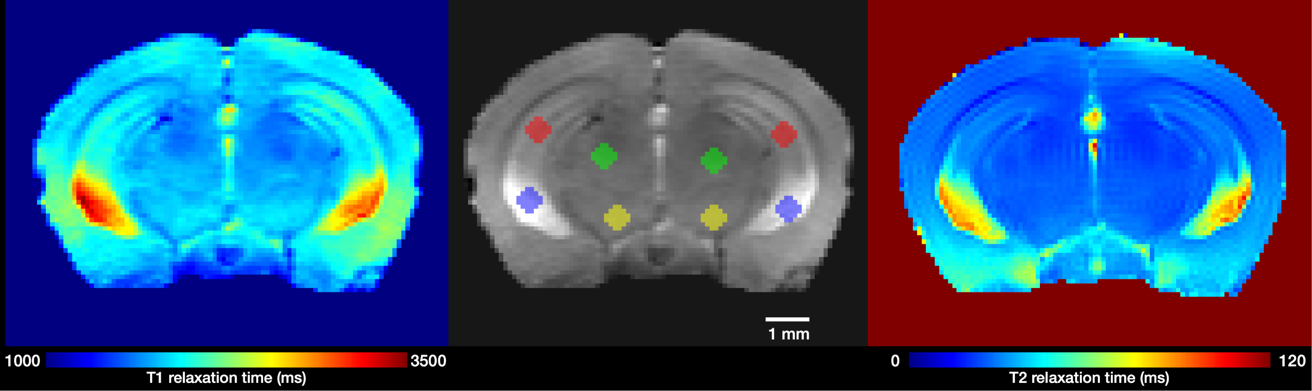 | | | |
| --- | --- | --- | --- |
| **Brain region/parameter** | **Control** | **HFD** | **P value** |
|  | **Mean ± SD (n=6)** | |  |
| Intracranial brain volume (mm^3^) | 481.92 ± 5.86 | 484.53 ± 9.46 | 0.5779 |
| Hippocampal volume (mm^3^) | 25.54 **±** 0.50 | 24.99 **±** 0.44 | 0.0676 |
| Ventricular volume (mm^3^) | 12.24 **±** 1.28 | 12.49 **±** 2.11 | 0.5887 |
|  | | | |
| Hippocampus/Brain volume ratio (%) | 5.30 **±** 0.14 | 5.16 **±** 0.08 | 0.0525 |
| Ventricles/Brain volume (%) | 2.58 **±** 0.44 | 2.54 **±** 0.26 | 0.8555 |
|  | | | |
| Hippocampal T1 relaxation time (ms) | 1943.83 ± 56.47 | 1900.29 ± 52.36 | 0.1961 |
| Hypothalamic T1 relaxation time (ms) | 1604.79 ± 105.8 | 1668.39 ± 45.88 | 0.2064 |
| Ventricular T1 relaxation time (ms) | 2577.25 ± 221.56 | 2276.53 ± 138.64 | 0.0182 |
|  | | | |
| Hippocampal T2 relaxation time (ms) | 41.38 **±** 1.21 | 41.50 **±** 0.79 | 0.8462 |
| Hypothalamic T2 relaxation time (ms) | 43.98 **±** 3.54 | 40.34 ± 2.74 | 0.0740 |
| Ventricular T2 relaxation time (ms) | 70.82 ± 9.35 | 59.57 ± 7.24 | 0.0420 |
| Thalamic T2 relaxation time (ms) | 36.56 **±** 0.4118 | 36.40 **±** 0.4905 | 0.8182 |

**Supplementary Table S2: Comparison of standard chow and high-fat diet (HFD).** This present HFD was chosen from among numerous available high-fat diets due to its frequent use in CNS research.

| **Diet Type** | **Provider** | **Fat** | **Carbohydrates** | **Protein** | **Density** |
| --- | --- | --- | --- | --- | --- |
| High fat diet (D12492) | Research Diets, Inc. | 60.0 kcal % | 20.0 kcal % | 20.0 kcal % | 5.21 kcal/g |
| Standard chow  (Safe^®^ D30) | SAFE^®^ Complete Care Competence | 14.1 kcal % | 60.0 kcal % | 26.0 kcal % | 3.34 kcal/g |

**Supplementary Table S3: List of tracers and injected concentration into cisterna magna and primary and secondary antibodies used for immunohistochemistry.**

| **Tracers** | | | | | | | | | | |
| --- | --- | --- | --- | --- | --- | --- | --- | --- | --- | --- |
| **Tracer** | | | **Solvent** | **Concentration (w/v)** | | | **Injection volume** | | **Manufacturer (CAT#)** | |
| Dextran, Fluorescein, 3000 MW, Anionic | | | aCSF | 1 % | | | 10 uL | | ThermoFisher Scientific (D3305) | |
| Ovalbumin, Alexa Fluor™ 647 Conjugate | | | aCSF | 1 % | | |  |  | ThermoFisher Scientific (O34784) | |
| **Primary antibodies** | | | | | | | | | | |
| **Antigen** | **Immunogen** | | | | **Manufacturer (CAT#)** | **Host & isotype** | | **Dilution** | | **Reference RRID#** |
| GFAP | Recombinant full length human GFAP isotype 1 expressed in and purified from E. coli. | | | | Thermo Fischer Scientific  PA1-1004 | Chicken / IgY | | 1:1000 | | AB_1074620 |
| AQP4 | C-terminus GST fusion protein with residues 249-323 of rat Aquaporin 4 (SwissProt accession number P47863). | | | | EMD Millipore  AB3594 | Rabbit | | 1:500 | | AB_91530 |
| CD68 | Purified Concanavalin A acceptor glycoprotein from P815 cell line. | | | | BIO-RAD MCA1957 | Rat IgG | | 1:100 | | AB_322219 |
| **Secondary antibodies** | | | | | | | | | | |
| **Antigen** | **Immunogen** | **Manufacturer (CAT#)** | | | **Host & isotype** | **Dilution** | | **Reference RRID#** | | **Fluorophore** |
| Chicken | Gamma Immunoglobins Heavy and Light chains | Thermeo Fischer Scientific  A11039 | | | Goat/ IgG | 1:500 | | AB_2534096 | | Alexa Fluor™ 488 |
| Rabbit | Gamma Immunoglobins Heavy and Light chains | Thermeo Fischer Scientific  A11011 | | | Goat/ IgG | 1:500 | | AB_143157 | | Alexa Fluor™ 568 |
| Rat | Gamma Immunoglobins Heavy and Light chains | Thermeo Fischer Scientific  A-21247 | | | Goat/ IgG | 1:500 | | AB_141778 | | Alexa Fluor™ 647 |
